# Incidence and Associated Risk Factors of Coeliac Disease in Children Newly Diagnosed With Type 1 Diabetes: A 5‐Year Nationwide Prospective Study From Kuwait

**DOI:** 10.1002/dmrr.70246

**Published:** 2026-10-28

**Authors:** Hessa Al‐Kandari, Wafaa Al‐Qabandi, Mohammed T. Hudda, Aisha Alsaqabi, Lujain D. AlAbduljaleel, Rawand Rafat Toma, Ahlam Mustafa, Fawaz Al‐Refaee, Dalia Al‐Abdulrazzaq

**Affiliations:** ^1^ Department of Population Health Dasman Diabetes Institute Kuwait City Kuwait; ^2^ Ministry of Health Kuwait City Kuwait; ^3^ Department of Pediatrics College of Medicine Kuwait University Safat Kuwait

**Keywords:** biopsy, internal medicine, medicine, paediatrics, prospective cohort study, risk factor, type 1 diabetes, younger age

## Abstract

**Aims:**

Kuwait has one of the highest global incidences of childhood type 1 diabetes (T1D), yet little is known about the burden of coeliac disease (CD) in this population. We aimed to determine the prevalence/incidence of CD in children newly diagnosed with T1D in Kuwait and identify associated risk factors.

**Methods:**

In this prospective cohort study, all 1938 children (≤ 14 years) diagnosed with T1D in Kuwait between 2011 and 2017 were enrolled and followed for 5 years post‐diagnosis. CD development was defined by intestinal biopsy confirmation. Cox univariable regression models quantified associations between several characteristics at T1D diagnosis (age, sex, DKA, height z‐score and BMI z‐score) and incident CD risk.

**Results:**

Four baseline CD cases were excluded. Of the remaining 1934 children, 65 (3.4%) developed CD during follow‐up. The median time to CD diagnosis was 1.5 years; 40% were diagnosed within the first year. The incidence rate within the first year following T1D diagnosis was 1.35 (95% CI 0.92–1.99) per 100 person‐years and generally declined over subsequent years of follow‐up. While age at diagnosis showed no clear effect shortly after T1D diagnosis, older age was associated with progressively lower CD risk over longer follow‐up, with the risk of CD being 33% lower per one‐year increase in age at diagnosis by 5 years of follow‐up; no other baseline factors investigated were significant.

**Conclusion:**

This nationwide prospective study highlights the burden and timing of biopsy‐confirmed CD after T1D onset. CD occurred most frequently during the early years after T1D diagnosis, with a higher risk among younger children, supporting particular attention to screening in these groups.

## 1 Introduction

Type 1 diabetes (T1D) is a chronic autoimmune condition characterised by the destruction of the insulin‐producing beta cells in the islets of Langerhans of the pancreas [1]. Given its autoimmune nature, individuals with T1D are at increased risk of developing other autoimmune conditions, including coeliac disease (CD), which may present with subtle or non‐specific symptoms [1, 2, 3, 4].

The coexistence of T1D and CD is well recognised, with multiple population‐based studies reporting prevalent cases of biopsy‐confirmed CD among children with T1D [5, 6]. In such cases, the dual diagnosis of CD in children with T1D increases the clinical burden, leading to additional dietary restriction, impaired growth, potential nutritional deficiencies, and adverse impact on the quality of life as well as glycaemic control of the individual, especially if CD remains undiagnosed [4, 7, 8, 9]. Given this established association and potential adverse consequences, screening patients with T1D for CD by serological testing enables early detection and timely management [10, 11, 12].

Kuwait ranks among the countries with the highest reported incidence of T1D in children, with rates that have more than doubled over the past 2 decades [13]. The most recent reported incidence of T1D in Kuwait was estimated to be 40.1 per 100,000 children aged 0–14 years according to the Childhood‐Onset Diabetes electronic Registry (CODeR), a national registry established in Kuwait since 2011 [13]. Despite this high burden, and the established link between T1D and incident CD risk, there has been no national data on the incidence or prevalence of CD in children with T1D in Kuwait. Data related to the incidence of CD among children with T1D is also lacking from other childhood settings globally. Additionally, little is known about potential factors associated with the development of CD in such a high‐risk population.

Therefore, this observational prospective study aims to address this gap by determining the prevalence and incidence of CD in children newly diagnosed with T1D in the country and identifying potential baseline characteristics associated with CD development in this population.

## 2 Materials and Methods

This observational prospective study enrolled all children newly diagnosed with T1D between 2011 and 2017 who were receiving follow‐up care at all governmental hospitals across Kuwait. In Kuwait, all children diagnosed with diabetes should be registered in CODeR, a nation‐wide diabetes registry with a high ascertainment rate [13, 14]. The follow‐up period extended for 5 years from the time of T1D diagnosis. Eligible participants were children diagnosed with T1D at ≤ 14 years of age who had documented data on CD screening and diagnosis during the follow‐up period.

Baseline data at T1D diagnosis were obtained through a review of the CODeR registry records as well as the medical records at the respective hospitals. The collected data included demographic characteristics, year of diagnosis, anthropometric measures, presentation with diabetic ketoacidosis (DKA) as per the International Society of Paediatric and Adolescent Diabetes (ISPAD) guidelines [15], Haemoglobin A1C (HbA1C), and CD screening and diagnosis. Anthropometric measures consisted of weight, height and body mass index (BMI) expressed in Z‐scores as per the World Health Organization (WHO) growth standards for children [16].

In governmental hospitals across Kuwait during the study period, screening for CD consisted of serological testing for tissue transglutaminase IgA antibodies (tTG‐IgA), provided total immunoglobulin A (IgA) levels were normal, and/or endomysial antibodies (EMA) were performed at the time of T1D diagnosis and annually thereafter. However, the diagnosis of CD in clinical practice during the study period was based exclusively on histopathological confirmation from intestinal biopsy. Serology‐based, no‐biopsy diagnostic criteria for selected children were introduced by ESPGHAN in 2012 [17], near the beginning of the study period. However, these criteria were not adopted into routine clinical practice by paediatric gastroenterologists in Kuwait during the study period, and biopsy remained the standard diagnostic requirement. Although CD serological testing was captured in the CODeR registry, documentation was incomplete during the study period and was sometimes limited to a positive or negative result without the corresponding antibody titre. Clinical features specific to CD and HLA data were also not routinely captured, precluding reliable retrospective application of serology‐based diagnostic criteria. In contrast, biopsy‐confirmed CD cases were comprehensively ascertained; therefore, case ascertainment was restricted to histologically confirmed CD to ensure diagnostic consistency and completeness. We conducted a rigorous, multi‐source verification process to ensure accurate identification of biopsy‐confirmed CD among children with T1D. This included a systematic review of medical and registry records as well as endoscopy lists obtained from paediatric gastroenterologists at the respective hospitals to identify children who underwent upper gastrointestinal endoscopy with concurrent T1D and histological confirmation of CD. Pathology laboratory records were cross‐checked to verify biopsy findings, and referrals to dietary services for gluten‐free diet support in children with confirmed CD diagnoses were also reviewed. This multi‐layered approach was applied consistently throughout the study period to optimise case capture and ensure diagnostic accuracy.

Statistical analysis was conducted using Stata v18.0. To assess the incidence of CD over the five‐year follow‐up period following the diagnosis of T1D, individuals diagnosed with CD prior to T1D diagnosis were removed from the analysis. Distributions of key continuous variables of age, weight, height, BMI z‐score, DKA, and HbA1c were investigated at baseline. Descriptive statistics of the mentioned key baseline variables were calculated, by incident CD diagnosis and overall, using median and interquartile ranges for continuous variables due to some skewness present for some of the variables. Categorical variables of gender and nationality group were described using counts and percentages.

For consistency with the study objective, the five‐year prevalence estimate was defined as the proportion of children without known CD at T1D diagnosis who were diagnosed with biopsy‐confirmed CD during the subsequent five‐year follow‐up. Annual incidence rate ratios, per 100 person‐years, for the development of CD up to the end of follow‐up were also calculated by sex, diabetes diagnosis age groups (0–4 years, 5–9 years, 10+ years) and overall. 95% Confidence Intervals for the incidence rates were estimated using the quadratic approximation to the Poisson log‐likelihood to account for small cell counts. Log‐rank tests were used to test for sex‐ and age‐group differences in the incidence of CD using a significance level of 95%. Kaplan‐Meier Survival curves were plotted by sex and diabetes diagnosis age groups.

Finally, we investigated whether there was an association between several factors at the time of T1D diagnosis, including age, sex, DKA score, and BMI z‐score, and incident CD diagnosis across the 5 years of follow‐up. These were explored using a series of univariable Cox proportional hazard regression models with CD as the outcome. The proportional hazard assumption was tested and in the event of a violation, models were refitted including the corresponding time‐varying covariate. To account for multiple testing, a Bonferroni correction was applied and a significance level of *p* = 0.0125 was used to ascertain statistical significance.

The study was performed in accordance with the Declaration of Helsinki and was approved by the Ethical Review Committee at the Dasman Diabetes Institute [RA 2015/010] and the Standing Committee for Coordination of Health and Medical Research at the Ministry of Health [651/2017].

## 3 Results

During the study period, 1938 children were newly diagnosed with T1D and registered in CODeR and had available data on CD screening and diagnosis at baseline and during follow‐up and were therefore included in the study. At T1D diagnosis, there were 4 prevalent cases of CD among the cohort of children included in the study and were thus excluded from analyses. Characteristics of the remaining 1934 children in the cohort at diagnosis of T1D are presented in Table 1. The median age at T1D diagnosis was 8.4 years (Interquartile range (IQR) 5.4, 10.7). The cohort was predominantly female (53.2%), with the vast majority of participants originating from the Middle East and North African (MENA) region (96.0%). Of the 1934 children followed up in the cohort, 65 (3.4%) developed CD. The median time to CD diagnosis was 1.5 years with almost half of those who developed CD (40%) were diagnosed within one‐year of diabetes diagnosis.

**TABLE 1:** Baseline characteristics of the cohort at diabetes diagnosis, overall and by incident coeliac disease diagnoses.

Variable	Median (LQ—UQ)/*n* (%)		
	Overall (*N* = 1934)	No CD (*n* = 1869)	CD (*n* = 65)
Age at type 1 diabetes diagnosis (years)	8.41 (5.35, 10.67)	8.50 (5.41, 10.70)	6.91 (4.13, 9.20)
Sex			
male	906 (46.8)	879 (47.0)	27 (41.5)
female	1028 (53.2)	990 (53.0)	38 (58.5)
Nationality group			
MENA	1856 (96.0)	1795 (96.0)	61 (93.8)
South Asian	48 (2.5)	46 (2.5)	2 (3.1)
Americas	14 (0.7)	13 (0.7)	1 (1.5)
European	6 (0.3)	5 (0.3)	1 (1.5)
African	1 (0.1)	1 (0.1)	0 (0.0)
Other/missing	9 (0.5)	9 (0.5)	0 (0.0)
DKA at type 1 diabetes diagnosis	529 (27.4)	507 (27.1)	22 (33.8)
Weight z‐score at type 1 diabetes diagnosis	0.24 (−0.68, 1.35)	0.23 (−0.71, 1.35)	0.29 (−0.50, 1.39)
Height z‐score at type 1 diabetes diagnosis	0.25 (−0.50, 1.07)	0.25 (−0.51, 1.07)	0.10 (−0.48, 0.94)
BMI z‐score at type 1 diabetes diagnosis	0.15 (−0.95, 1.54)	0.15 (−0.95, 1.54)	0.05 (−0.99, 1.64)
HbA1c at type 1 diabetes diagnosis	11.2 (9.7, 12.7)	11.2 (9.7, 12.7)	11.0 (10.0, 12.3)

*Note: p*‐value from non‐parametric test of differences in medians (continuous variables) or test of equality of proportions (categorical variables).

Abbreviations: BMI, body mass index; CD, coeliac disease; DKA, diabetic ketoacidosis; HbA1c, haemoglobin A1c.

The incidence rate, per 100 person‐years, was calculated per year of follow‐up and is presented by sex and overall in Table 2 and by baseline age group in Table 3. The incidence rate within the first year following T1D diagnosis was 1.35 (95% CI 0.92–1.99) per 100 person‐years and generally declined over subsequent years of follow‐up. There was a statistically significant difference in the incidence rates across age groups (p_log‐rank_ = 0.02), but not by sex (p_log‐rank_ > 0.05). While overall incidence rates across all three age groups had overlapping 95% confidence intervals, incidence decreased with increasing age at T1D diagnosis (0.88, 0.59, and 0.35 per 100 person‐years for ages 0–4, 5–9, and ≥ 10 years, respectively). Furthermore, almost all incident cases of CD amongst the oldest age group were diagnosed less than 2 years post‐T1D diagnosis, although the number of cases in this age group was small (Table 3). These patterns are demonstrated graphically in the Kaplan‐ Meier curves of Coeliac Disease‐free survival over 5 years of follow‐up, stratified by sex (top) and T1D diagnosis age groups (bottom) Figure 1.

**TABLE 2:** Incidence rates of coeliac disease, per 100 person‐year, for 5 years of follow‐up post diagnosis of type 1 diabetes by sex and overall.

Years post type 1 diabetes diagnosis	Males			Females			Overall
	No. of cases	Person‐years	Incidence rate per 100 person‐years (95% CI)	No. of cases	Person‐years	Incidence rate per 100 person‐years (95% CI)	
Under 1 year	8	900.51	0.89 (0.44–1.78)	18	1018.98	1.77 (1.11–2.80)	1.35 (0.92–1.99)
1st	5	896.41	0.56 (0.23–1.34)	6	1005.90	0.60 (0.27–1.33)	0.58 (0.32–1.04)
2nd	5	889.86	0.56 (0.23–1.35)	6	1000.33	0.60 (0.27–1.34)	0.58 (0.32–1.05)
3rd	6	884.84	0.68 (0.30–1.51)	3	995.81	0.30 (0.10–0.93)	0.48 (0.25–0.92)
4th	2	881.56	0.23 (0.06–0.91)	1	994.37	0.10 (0.01–0.71)	0.16 (0.05–0.50)
5th	1	879.36	0.11 (0.02–0.81)	4	992.14	0.40 (0.15–1.07)	0.27 (0.11–0.64)
Overall	27	5332.54	0.51 (0.35–0.74)	38	6007.55	0.63 (0.46–0.87)	0.57 (0.45–0.73)

**TABLE 3:** Incidence rates of coeliac disease, per 100 person‐years, for 5 years of follow‐up post diagnosis of type 1 diabetes, by diabetes diagnosis age groups and overall.

	No. of cases	Person‐years	Incidence rate per 100 person‐years (95% CI)
0–4 years			
Under 1 year	4	425.77	0.94 (0.35–2.50)
1st	3	421.21	0.71 (0.23–2.21)
2nd	4	418.21	0.96 (0.36–2.55)
3rd	5	413.23	1.21 (0.50–2.91)
4th	2	409.93	0.49 (0.12–1.95)
5th	4	407.14	0.98 (0.37–2.62)
Overall	22	2495.50	0.88 (0.58–1.34)
5–9 years			
Under 1 year	14	860.40	1.63 (0.96–2.75)
1st	4	852.45	0.47 (0.18–1.25)
2nd	7	844.98	0.83 (0.39–1.74)
3rd	3	841.41	0.36 (0.11–1.11)
4th	1	840.00	0.12 (0.02–0.85)
5th	1	838.36	0.12 (0.02–0.85)
Overall	30	5077.59	0.59 (0.41–0.85)
10+ years			
Under 1 year	8	632.31	1.27 (0.63–2.53)
1st	4	627.66	0.64 (0.24–1.70)
2nd	0	626.00	0.00 (0.00–0.00)
3rd	1	625.01	0.16 (0.02–1.14)
4th	0	625.00	0.00 (0.00–0.00)
5th	0	625.00	0.00 (0.00–0.00)
Overall	13	3760.99	0.35 (0.20–0.60)

**FIGURE 1:**
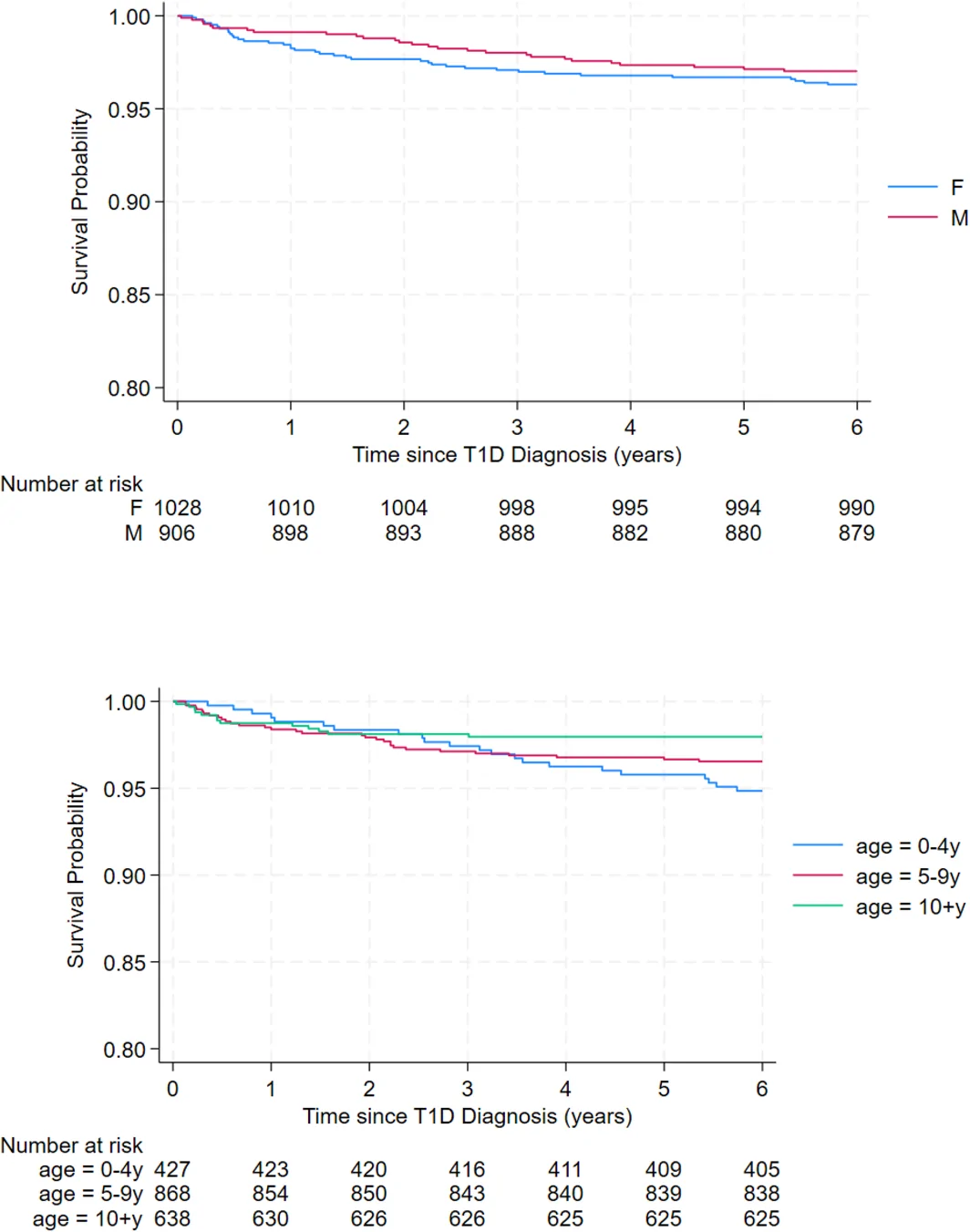
Kaplan‐Meier curves of coeliac disease‐free survival over 5 years of follow‐up, stratified by sex (top) and diabetes diagnosis age groups (bottom).

Figure 2 presents the results of the univariable associations between several baseline characteristics and the incidence of CD. The proportional hazards assumption for baseline age was violated (*p* < 0.001), indicating a time‐varying association, with age showing little association with CD risk shortly after T1D diagnosis, while older age was associated with progressively lower CD risk over longer follow‐up (Figure 2A). No other baseline characteristics investigated (sex, DKA, Height z‐score or BMI z‐score) showed associations with the incidence of CD (Figure 2B).

**FIGURE 2:**
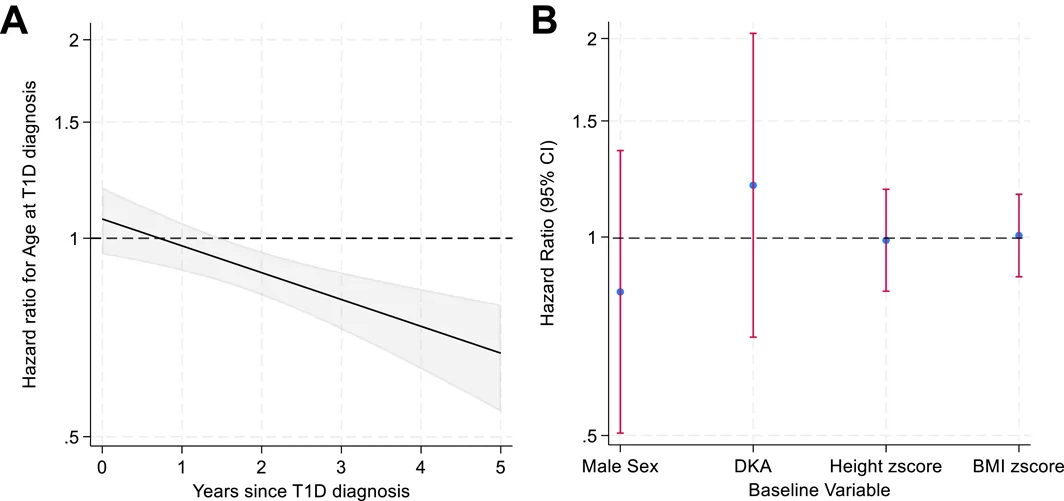
Univariable associations between baseline characteristics at type 1 diabetes diagnosis and incident diagnosis of coeliac disease. (A)—Estimated hazard ratios for the association between age at diagnosis and risk of incident coeliac disease are plotted against time since type 1 diabetes (solid line) diagnosis due to a violation of the proportional hazards assumption. Therefore, a time‐varying coefficient (age × time) was included in the Cox proportional hazards model. (B)—Estimated hazard ratios for the associations between several baseline variables at the time of T1D diagnosis and the risk of incident coeliac disease. BMI, body mass index; DKA, diabetic ketoacidosis.

## 4 Discussion

This study represents the first report from Kuwait to investigate the development of CD among children newly diagnosed with T1D. We report on the five‐year annual incidence rate, overall incidence and prevalence, as well as the baseline characteristics associated with CD development in this high‐risk population. Age at T1D diagnosis was associated with subsequent CD in a time‐dependent manner. The effect was weak early after T1D diagnosis but strengthened over time, such that younger age at T1D diagnosis was associated with higher CD risk during longer follow‐up.

The strong association between T1D and CD is supported by large population‐based data; in a UK cohort of over 22 million individuals, childhood‐onset T1D was associated with a 28‐fold higher rate of CD compared with the general population [18]. The well‐established association between T1D and CD is partly explained by substantial shared genetic susceptibility. Both diseases are strongly associated with HLA class II haplotypes, particularly HLA‐DR3‐DQ2, while HLA‐DR4‐DQ8 also contributes to their overlapping susceptibility [19]. Beyond HLA, large genetic analyses have identified several shared non‐HLA susceptibility loci, including RGS1, IL18RAP, TAGAP, PTPN2, CTLA4, and SH2B3, supporting common autoimmune pathways underlying both conditions [20].

Few studies have reported the incidence rate of the development of CD in the paediatric population diagnosed with T1D. One report by Vajravelu et al. from a sample of patients with T1D registered in the Health Improvement Network (THIN), a United Kingdom primary care database [21], reported an overall same incidence rate of 0.57 per 100 person‐years amongst their sample of children < 18 years of age [21]. However, when they restricted their analyses to children aged ≤ 10 years old, the reported incidence was higher than in our study (1.45 per 100 person‐years) [21]. The report also noted a higher incidence in females; however, in our cohort, no significant association between sex and CD development was observed [21]. The majority of studies on the development of CD in children with T1D are cross‐sectional, focussing primarily on prevalence. Our observed prevalence of biopsy‐confirmed CD (3.4%) aligns within the lower to mid‐range of regional and international estimates. This prevalence was close to that reported in a meta‐analysis encompassing over 300,000 patients across 87 studies, which estimated a pooled prevalence of CD among individuals with T1D at 4.5% [22]. An Omani cohort reported a slightly higher CD prevalence of 5.5% in children with T1D [23], while studies from Saudi Arabia and Jordan reported biopsy‐confirmed prevalences of 7.5% and 9.1%, respectively [11, 24]. Internationally, the SWEET registry reported an overall CD prevalence of 4.5% with regional variation [25], while studies from Sweden, the US T1D Exchange, Germany/Austria DPV, U.K. NPDA, and Australian ADDN registries reported prevalences ranging from 1.9% to 9.8% [26, 27]. The US SEARCH for Diabetes in Youth study reported diagnosed CD in 7% of 2444 youth with T1D, with an additional proportion having serological evidence of potentially undiagnosed CD [28]. Although close to some international reports, the study reports a lower rate of CD in children with T1D in comparison to neighbouring countries in the region. This finding is particularly notable given that Kuwait ranks among the countries with the highest incidence of T1D globally and within the Middle East region [13]. Despite this high burden of T1D, the prevalence of biopsy‐confirmed CD in the study is relatively lower than that reported by regional counterparts. Nevertheless, these comparisons should be interpreted with caution, as many of the available studies differ methodologically from our study. In particular, the majority are cross‐sectional rather than prospective in design, and few have specifically examined children from the time of T1D diagnosis. Furthermore, unlike some studies reporting overall CD prevalence in children with T1D, our study excluded children with CD diagnosed before T1D diagnosis. The prevalence of CD in children with T1D reported in our study might reflect a generally lower prevalence of CD in the Kuwaiti population. Although, population‐based studies investigating the true prevalence or incidence of CD in Kuwait are scarce, Al‐Qabandi et al. [29] reported a biopsy‐confirmed CD incidence rate of 6.9 per 100,000 among symptomatic and screened children referred to gastroenterology centres in Kuwait. However, due to differences in study methodology and in the calculation of incidence rates, direct comparisons between the incidence of CD reported in the general paediatric population by Al‐Qabandi et al. [29] and the incidence observed in our cohort of children with T1D are not appropriate. Consequently, it cannot be directly concluded that the incidence of CD in children with T1D in our study reflects the general incidence rate of CD in the country. However, Al‐Qabandi et al. [29] had discussed that the incidence of CD reported in the general paediatric population in Kuwait was lower in comparison to neighbouring countries [29, 30, 31]. Broader population trends in CD incidence/prevalence may additionally influence the rates observed in specific high‐risk groups. However, variability in screening and biopsy practices may also contribute, particularly in children with T1D, where concerns regarding diagnostic burden, dual diagnosis, and invasive procedures may influence clinical decisions and family acceptance of biopsy confirmation.

In our cohort, the median time to diagnosis with CD was 1.5 years and 40% of the patients developed CD within the first year after diagnosis with T1D. Our findings are consistent with evidence from a meta‐analysis and multiple longitudinal studies showing that a substantial proportion of CD cases develop within the first year after T1D diagnosis [22]. We observed a similar median time to CD diagnosis as an Italian cohort (1.3 years) [32], but shorter than reports from the UK (2.1 years) [21], Sweden (2.7 years) [26], and France (4.1 years) [33]. Likewise, the SWEET registry and other international cohorts reported that many CD cases are diagnosed within the first one to 2 years following T1D diagnosis [25, 34], supporting current recommendations emphasising intensified screening during the early years after T1D onset [5]. The observed clustering of CD diagnoses early after T1D onset should be interpreted cautiously, as some asymptomatic children may have had undiagnosed CD before diabetes diagnosis and were identified only through subsequent diabetes‐related screening, consistent with population‐screening data demonstrating a substantial burden of previously unrecognised CD autoimmunity in asymptomatic children [4, 35, 36]. Thus, the early time‐course may partly reflect detection of previously unrecognised disease rather than new CD development, particularly in Kuwait, where routine CD screening is primarily undertaken in children with T1D rather than the general paediatric population.

In our study, the association between age at T1D diagnosis and biopsy‐confirmed CD was time‐dependent, becoming more pronounced with longer follow‐up, with younger children increasingly more likely to develop CD. Our finding is consistent with large international registry data, in which children with coexisting CD were significantly younger at T1D diagnosis than those with T1D alone [27]. Likewise, An Italian multicenter cohort study published in 2004 involving 4322 children demonstrated that younger age at the onset of T1D was significantly associated with an increased risk of developing CD [32]. Two decades later, a 2024 publication reaffirmed that early age at T1D diagnosis remains a strong predictor of CD development during childhood [26]. The association between younger age at T1D diagnosis and subsequent CD may also reflect the biological heterogeneity of T1D, as younger‐onset disease is characterised by a more aggressive autoimmune phenotype and may carry genetic profiles that confer greater susceptibility to concurrent autoimmunity, including CD [37, 38].

In contrast to some published studies, our analysis did not identify a significant association between gender and the risk of CD development. Previous research has identified female gender as a potential risk factor, with findings from the Italian multi‐centre study indicating a higher incidence of CD among female children with T1D [32]. Furthermore, broader literature demonstrates a female predominance in CD, particularly during childhood [39, 40]. In our study, the incidence of CD was higher in females only in the first year after T1D diagnosis. However, using the regression model, gender was not associated with the development of CD in children newly diagnosed with T1D in Kuwait. The absence of a gender effect in our cohort might suggest that gender‐specific associations may vary across populations and emphasises the importance of context‐specific evaluation in future research.

This study has several notable strengths. First, it is a nationwide, population‐based study conducted in a country with one of the highest reported incidence rates of T1D globally and utilising a well‐established registry with a high ascertainment rate [13, 14]. The study's prospective, longitudinal design allowed for consistent follow‐up of children from the time of T1D diagnosis, enabling an accurate assessment of the incidence and timing of CD development within a uniform clinical and environmental context. Furthermore, while the number of CD cases annually was low, we calculated the confidence intervals for the incidence rates using the quadratic approximation to the Poisson log‐likelihood to ensure we accurately estimated the level of imprecision. Additionally, data collection was performed using a standardised registry data sheet, enhancing consistency and reliability across reporting centres. Another key strength is the high diagnostic rigour, with biopsy‐confirmed diagnosis of CD applied uniformly across all participating centres, ensuring specificity and minimising diagnostic misclassification, supported by a rigorous multi‐source ascertainment process that included review of registry records, hospital medical records, endoscopy lists, pathology reports, and dietary referrals.

However, several limitations should be acknowledged. As an observational study, the analysis may be affected by residual confounding from unmeasured factors. Additionally, reliance on biopsy confirmation may have missed children whose families declined the procedure, potentially underestimating CD incidence and prevalence; as the number of biopsy refusals was not routinely captured within the CODeR registry. Furthermore, the registry did not capture detailed information on socioeconomic status, perinatal history, dietary patterns, COVID‐19 exposure or infection status, or specific environmental exposures, all of which may influence CD development in high‐risk populations. Finally, data on HLA genotypes and family history of CD and other autoimmune diseases, both recognised risk modifiers that may help explain inter‐individual variability in CD development, are not part of the CODeR registry data.

In conclusion, this first nationwide study from Kuwait reports the five‐year prevalence and incidence of biopsy‐confirmed CD in children after T1D diagnosis and identifies younger age at T1D diagnosis as a significant risk factor for CD development during follow‐up. While children with T1D remain at risk throughout life, our findings support intensified screening during the early years after T1D diagnosis, with particular attention to children diagnosed with T1D at a younger age, in addition to continued long‐term surveillance.

Our findings describe the timing of CD diagnosis during 5 years of follow‐up but do not define an evidence‐based point at which screening can safely be discontinued. Decisions regarding the frequency and duration of CD screening should therefore remain guided by established clinical recommendations. Continued prospective surveillance and registry‐based research are needed to better define longer‐term risk and to identify genetic, immunological, and environmental determinants that may support more individualised screening strategies.

## Author Contributions

D.A. served as the principal investigator and conceptualised, planned, executed the study, and drafted the manuscript. M.T.H. conducted data analysis and management and contributed to writing the original draft. W.Q. contributed to patient data management and drafting the manuscript. A.S. contributed to writing the original draft and was involved in reviewing and editing the manuscript. L.D.A. contributed to the writing of the manuscript. R.R.T. and A.M. were involved in patient data management and contributed to drafting the manuscript. F.R. was involved in patient data management. H.K. contributed to study planning, execution, and patient data management. All authors reviewed and approved the final version of the manuscript. D.A. is the guarantor of this work, with full access to all study data, and assumes responsibility for the integrity and accuracy of the data analysis.

## Funding

This project was funded by the Dasman Diabetes Institute, Kuwait City, Kuwait [RA 2015/010].

## Ethics Statement

The study was performed in accordance with the Declaration of Helsinki and was approved by the Ethical review committee at Dasman Diabetes Institute [RA 2015/010] and the Standing Committee for Coordination of Health and Medical Research at the Ministry of Health [651/2017]. The data used in this study were completely anonymised without any possibility of re‐identifying subjects. A waiver of consent was granted by the Standing Committee for Coordination of Health and Medical Research at the Ministry of Health.

## Conflicts of Interest

The authors declare no conflicts of interest.

## Data Availability

The data that support the findings of this study are available from the Ministry of Health of Kuwait (MOH) and the Dasman Diabetes Institute, but restrictions apply to the availability of these data, which were used under license for the current study and therefore are not publicly available. Data are however available from the authors upon reasonable request and with permission from the MOH.
